# Survey on the perceptions of UK gastroenterologists and endoscopists to artificial intelligence

**DOI:** 10.1136/flgastro-2021-101994

**Published:** 2022-01-17

**Authors:** Rawen Kader, Rebecca F Baggaley, Mohamed Hussein, Omer F Ahmad, Nisha Patel, Gareth Corbett, Sunil Dolwani, Danail Stoyanov, Laurence B Lovat

**Affiliations:** 1 Division of Surgery and Interventional Sciences, University College London, London, UK; 2 Wellcome/EPSRC Centre for Interventional and Surgical Sciences (WEISS), University College London, London, UK; 3 Department of Gastroenterology, University College London Hospitals NHS Foundation Trust, London, UK; 4 Department of Respiratory Infections, University of Leicester, Leicester, UK; 5 Department of Gastroenterology, Imperial College Healthcare NHS Trust, London, UK; 6 Department of Gastroenterology, Addenbrooke's Hospital, Cambridge, UK; 7 Division of Population Medicine, School of Medicine, Cardiff University, Cardiff, UK

**Keywords:** computerised image analysis, endoscopy

## Abstract

**Background and aims:**

With the potential integration of artificial intelligence (AI) into clinical practice, it is essential to understand end users’ perception of this novel technology. The aim of this study, which was endorsed by the British Society of Gastroenterology (BSG), was to evaluate the UK gastroenterology and endoscopy communities’ views on AI.

**Methods:**

An online survey was developed and disseminated to gastroenterologists and endoscopists across the UK.

**Results:**

One hundred four participants completed the survey. Quality improvement in endoscopy (97%) and better endoscopic diagnosis (92%) were perceived as the most beneficial applications of AI to clinical practice. The most significant challenges were accountability for incorrect diagnoses (85%) and potential bias of algorithms (82%). A lack of guidelines (92%) was identified as the greatest barrier to adopting AI in routine clinical practice. Participants identified real-time endoscopic image diagnosis (95%) as a research priority for AI, while the most perceived significant barriers to AI research were funding (82%) and the availability of annotated data (76%). Participants consider the priorities for the BSG AI Task Force to be identifying research priorities (96%), guidelines for adopting AI devices in clinical practice (93%) and supporting the delivery of multicentre clinical trials (91%).

**Conclusion:**

This survey has identified views from the UK gastroenterology and endoscopy community regarding AI in clinical practice and research, and identified priorities for the newly formed BSG AI Task Force.

**Significance of this study**

**What is already known on this topic?**

- There is limited knowledge of the perspective of end users in gastroenterology/endoscopy to artificial intelligence (AI) technology. To date, this has only been explored in the US gastroenterology community.

**What this study adds?**

- Quality improvement in endoscopy (97%) was perceived to be the most significant benefit in applying AI to clinical practice, while the most significant challenges were accountability for the incorrect diagnoses (85%) and bias of algorithms (82%). Participants consider the priorities for the British Society of Gastroenterology (BSG) AI Task Force to be identifying research priorities (96%), guidelines for adopting AI devices in clinical practice (93%) and supporting the delivery of multicentre clinical trials (91%).

**How might it impact on clinical practice in the foreseeable future?**

- The survey results provide an insight into the views of UK gastroenterologists and endoscopists to AI technology. The findings can help propel the specialty forward in the clinical translation of AI, identify priorities for the newly formed BSG AI Task Force and hopefully improve patient care and outcomes.

## Introduction

Artificial intelligence (AI) is the ability of computers to perform tasks that traditionally require human intelligence such as learning and problem-solving.^1^ Several studies in the last few years have demonstrated the potential of AI applied to gastroenterology, with the specialty leading the way for randomised controlled trials (RCTs).^2–4^ Recent advancements in AI are primarily due to three main factors: (1) improvements in the accessibility of computational powers of graphic processing units; (2) advancement of algorithms using techniques such as deep learning, which allow automated learning from data; (3) increased availability of public datasets.^5^

There are now multiple regulatory-approved AI systems available in the market to aid colonic polyp detection and characterisation and the identification of dysplasia in Barrett’s oesophagus. New technologies are being developed including automated interpretation of video capsule images, determining the severity of mucosal inflammation in inflammatory bowel disease and a host of non-endoscopic AI tools using natural language programming. With the likely integration of AI into clinical practice, it is essential to understand the end user perception of this novel technology. To date, this has only been explored in the US gastroenterology community.^6 7^

The aim of this study, which was endorsed by the British Society of Gastroenterology (BSG), was to survey the UK gastroenterology and endoscopy community to assess their views on the benefits and barriers to the clinical application of AI technology, research in AI and the priorities for the newly established BSG AI Task Force.

## Methodology

### Study design

This is a prospective cross-sectional observational study. Participants from diverse clinical roles (consultants, trainees and clinical endoscopists) and workplace environments (secondary care, tertiary care and university academic departments) were recruited to complete an online survey.

For the survey’s conceptualisation of themes, we created a focus group of five experts in the field of AI in gastroenterology. Themes and question items were identified by consensus between members of the focus group. Junior and senior gastroenterologists undertook pilot testing at a teaching hospital in London. Minor adaptions were made before the survey was finalised.

Questions used 3-point and 5-point Likert scales and multiple-choice questions. Five themes were explored: (1) participant demographics, (2) participant experience in AI, (3) benefits and barriers of adopting AI in clinical practice, (4) priorities of and barriers to research in AI, and (5) priorities for the BSG AI Task Force. It was mandatory to complete the questions for themes (1) and (2) while the remaining ones were optional.

### Data collection

The survey was distributed via multiple methods over a period of 5 months (October 2020–February 2021). All BSG members were invited to complete the survey via the electronic BSG Newsletter, which advertised the survey weblink. The newsletter was emailed to 3154 members and was opened by 1268. Additionally, the survey weblink was disseminated via email to members of the BSG AI Task Force. The survey weblink was also available on the BSG Open Survey webpage, which is accessible to BSG and non-BSG members. Participants were encouraged to invite colleagues to complete the survey.

All participants provided electronic consent at the start of the survey. Participants entered their responses online using Research Electronic Data Capture tools hosted on the University College London Data Safe Haven.

### Statistical analysis

Descriptive statistics were used to summarise the survey results. Categorical data were reported as proportions (percentages) and analysed through cross-tabulation statistics using the Χ^2^ test (or Fisher’s exact test, where appropriate). A p value of <0.05 indicates statistical significance. All statistical calculations were performed using GraphPad Prism software (San Diego, California, USA), V.9.0.0, while graphs were constructed using Microsoft Excel V.16.48 and RStudio V.1.1.1106.

## Results

A total of 104 participants completed the survey.

### Demographic characteristics of participants

Participants (n=104) were spread across 3 countries (England, Wales and Scotland) and 11 regions, with the majority from London (63%) (online supplemental figure 1). Participants included 54 consultants (52%), 41 trainees (39%) and 9 clinical endoscopists (9%), with the latter referring to endoscopists from a nursing background. The primary place of work was tertiary care centres (52%), followed by secondary care (33%) and university academic departments (15%). Most participants had more than 5 years of experience in their specialty (69%), and almost half had more than 10 years of experience (43%) (online supplemental figure 2). The participants with less than 5 years of experience in their specialty (31%) consisted of 4 clinical endoscopists and 28 registrars.

10.1136/flgastro-2021-101994.supp2 Supplementary data

10.1136/flgastro-2021-101994.supp3 Supplementary data

### Participants’ experience of AI

Most of the participants had no formal education or qualification in AI (72%), which we defined as either attendance at an organised AI teaching day (25%) or completion of an AI course with certification (3%). Almost half of participants rated themselves as only ‘slightly familiar’ (47%) with research methodology in AI, 26% as ‘not familiar at all’, 19% ‘moderately familiar’ and 8% as ‘very familiar’. A similar proportion (45%) had read less than 5 AI-related papers, with 25% having read 5–20, 15% none and 14% more than 20.

### Perceptions to the clinical application of AI

Participants (n=92) perceived the most significant benefits in the application of AI to be in quality improvement of endoscopy (97%) and better endoscopic diagnosis (92%) (online supplemental figure 3). Non-consultants (trainees and clinical endoscopists) were more likely to believe the most significant benefit to be in faster endoscopy times compared with consultants (53% vs 24%; p<0.01).

10.1136/flgastro-2021-101994.supp4 Supplementary data

**Figure 1:**
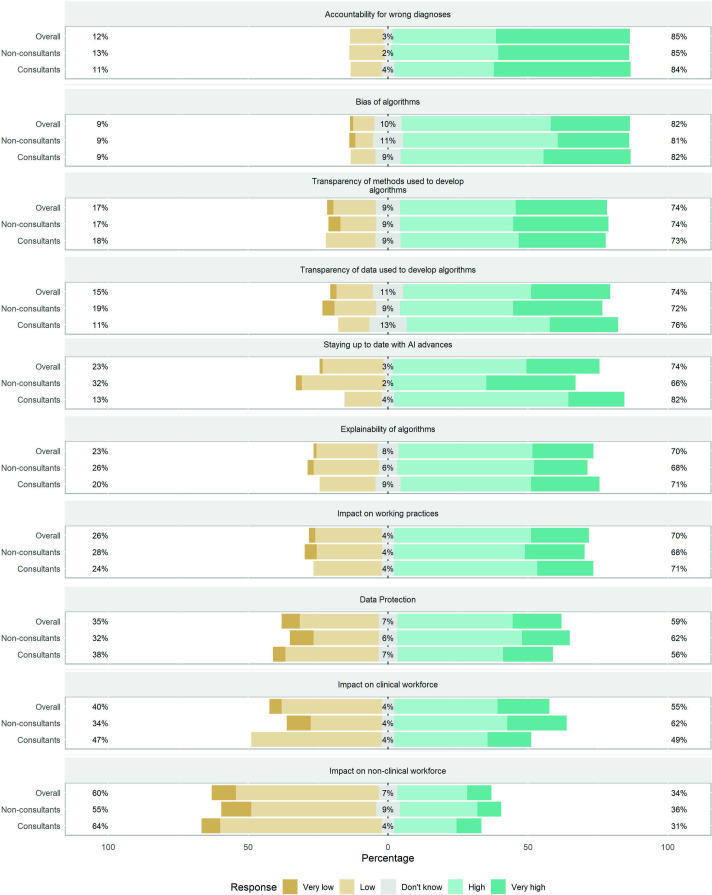
Participants’ response to the greatest challenges of using artificial intelligence (AI) in clinical practice.

The most significant perceived challenges of using AI were accountability for incorrect diagnoses (85%) and bias of algorithms (82%) (figure 1). Consultants demonstrated greater concern than non-consultants regarding the challenge of remaining up to date with AI advances (82% vs 66%, p<0.05). Participants who rated themselves as familiar (moderate to very) with AI research methods were more likely to be concerned with the transparency of the methods used to develop algorithms (87% vs 65%, p<0.02) and the data used to develop algorithms (92% vs 61%, p<0.03).

**Figure 2:**
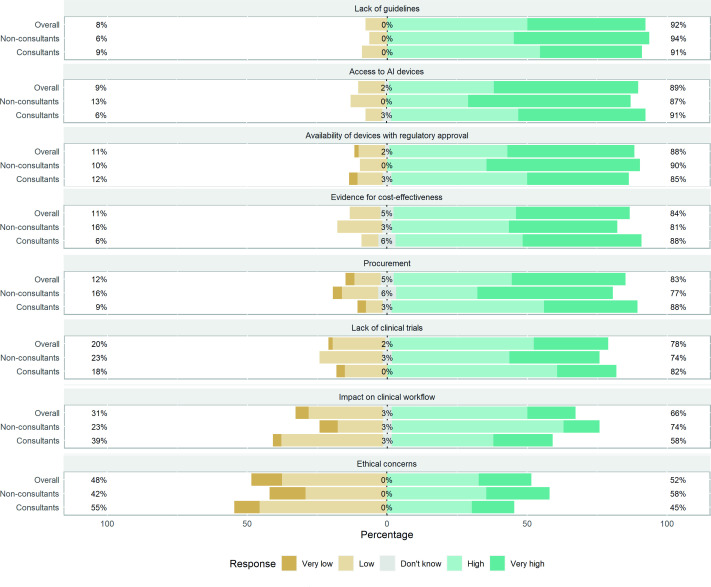
Participants’ response to the greatest barriers of adopting artificial intelligence (AI) in clinical practice.

Participants (n=64) viewed the most significant barriers to adopting AI in routine clinical practice to be a lack of guidelines (92%) and local access of the hospital to AI devices (89%) (figure 2). Those identifying themselves as familiar with research methods in AI held a greater belief that local access to AI devices poses a greater barrier to adoption of AI when compared with those less familiar (not at all to slightly) (97% vs 82%: p<0.03).

### Perceptions about research in AI

Participants (n=60) expressed that the priority of AI research should be in real-time endoscopic image diagnosis (95%) followed by general quality improvement (87%), automated reporting (82%) and lastly, natural language processing (NLP) (70%) (online supplemental figure 4).

10.1136/flgastro-2021-101994.supp5 Supplementary data

For AI research in endoscopy, 92% of participants ranked colonoscopy as a very high/high priority, followed by the upper gastrointestinal system (UGI) (67%) and capsule endoscopy (35%). Participants in secondary care viewed research in the UGI system as a higher priority than tertiary care colleagues (85% vs 53%, p<0.05).

**Figure 3:**
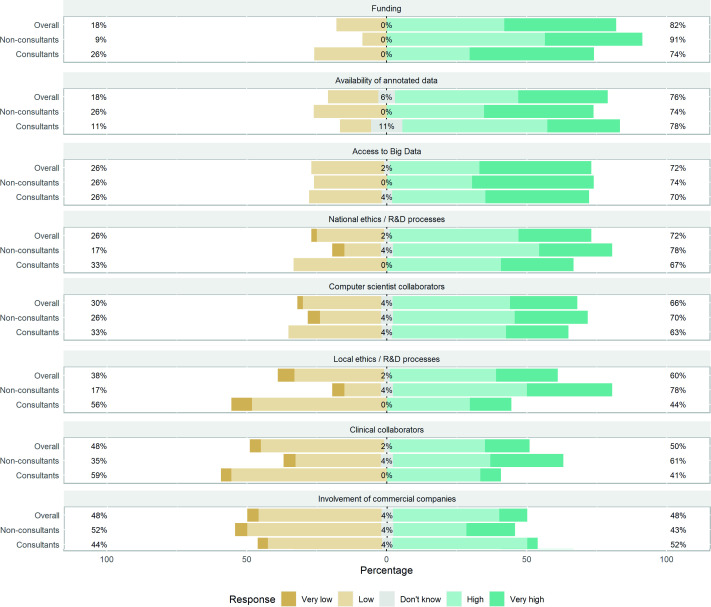
Participants’ response to the greatest barrier to artificial intelligence research in gastroenterology. R&D, research & development.

Participants (n=54) perceived the most significant barriers to AI research to be funding (82%), the availability of annotated data (76%) and access to Big Data (72%) (figure 3). Non-consultants were more likely than consultants to view local ethical/research and development processes as a greater barrier to AI research (78% vs 44%, p<0.01).

### Priorities for the BSG AI Task Force

**Figure 4:**
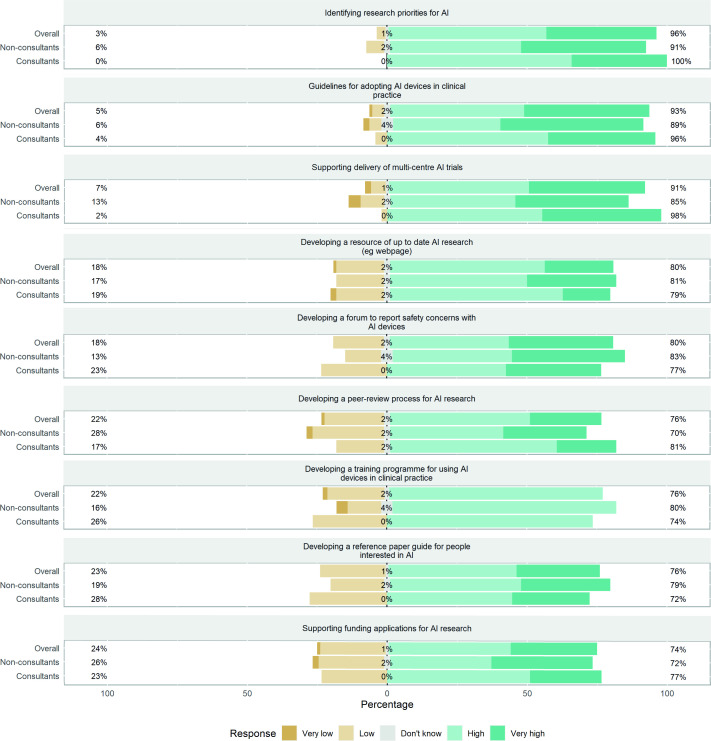
Participants’ response to the priorities for the British Society of Gastroenterology AI Task Force. AI, artificial intelligence.

The main priorities for the BSG AI Task Force (n=94) ascertained through this survey were to identify research priorities (96%), develop guidelines for adopting AI devices in clinical practice (93%) and support the delivery of multicentre clinical trials (91%) (figure 4). Participants with more than 10 years of experience in their specialty were more likely to prioritise supporting the delivery of multicentre AI trials (100% vs 85%, p<0.02) and funding applications for AI research (88% vs 64%, p<0.02). A greater proportion of participants who self-rated themselves as less familiar with AI research were more likely to view developing a resource of up-to-date AI research (eg, webpage) as a higher priority than those more familiar; however, this did not reach statistical significance (87% vs 69%, p=0.0539).

## Discussion

We report the results of the first survey to evaluate the perceptions of the UK gastroenterology and endoscopy community to AI.

Quality improvement of endoscopy and endoscopic diagnosis was the greatest perceived benefit of AI to clinical practice. This may reflect the greater familiarity with AI’s application to endoscopy. Currently, all AI-related RCTs, human benchmarking studies and regulatory-approved technologies within gastroenterology are in their application to endoscopy.^2 3 8 9^ Despite the lack of long-term longitudinal studies to evaluate the effect of AI on interval cancers, which are currently ongoing, there is an apparent belief among participants that it will be beneficial to endoscopic practice.^10 11^

Accountability for incorrect diagnoses and bias of algorithms were identified as the main challenges of using AI. The issue of accountability for wrong diagnoses is a complex problem. Errors will almost certainly occur with AI, and the cause of errors resulting in patient harm may differ in different scenarios.^12^ Further complicating this, regulatory-approved AI models currently function as a second reader, but this may change as training datasets grow and more robust algorithms are developed. This result emphasises the need for clarification around liability in the event of misdiagnoses. Regarding bias, the data used to train AI algorithms determine the quality of the model, and careful consideration in curating datasets and multicentre evaluation is required to protect against inherent biases in data which can result in models generalising poorly to the broader population and discriminating against certain diseases or patient demographics.^13^ Participants who rated themselves as familiar with AI research methods were more likely than those less familiar to be concerned with the transparency of the methods and data used to develop algorithms. Transparency is essential to help determine weaknesses and limitations of algorithms which helps to identify their appropriate application in the clinical workflow.^13^ Several key reporting guidelines are currently being upgraded, which should improve the transparency of reporting in AI studies.^14–16^

Despite the availability of regulatory-approved AI technology, its adoption into routine clinical practice in endoscopy has been slow. A lack of guidelines was identified as the main barrier to its adoption, and addressing this would help drive the specialty forward in the clinical translation of AI. Local access of hospitals to AI technology was another important barrier. Careful consideration and planning are required by national organisations such as the National Institute for Health and Care Excellence, NHSx and the BSG to avoid a two-tier healthcare system emerging in the National Health Service.

Respondents expressed that the priority of AI research should be in endoscopic image diagnosis followed by quality improvement. Ninety-two per cent of participants ranked colonoscopy as the highest priority for AI research, followed by 67% for UGI endoscopy, with participants in secondary care prioritising the UGI system more than tertiary care colleagues. This suggests that endoscopic diagnosis of the UGI system using AI may play a more significant role in secondary care and should be explored further.

Participants perceive the most significant barriers to AI research to be funding, the availability of annotated data and access to ‘Big Data’. Funding is an issue that is broadly applicable to all research, whereas the availability of annotated data and access to Big Data is more specific to AI.^17^ Human annotation is time-consuming and expensive, but novel platforms, such as Cord Vision, are emerging to improve the efficiency of annotating.^18^ Access to ‘Big Data’ remains an issue with academics and industry primarily relying on small private datasets to train algorithms due to the limited number of public datasets in gastroenterology. In contrast to this, specialities such as radiology have access to open-source datasets of radiographs that exceed 100 000 patients.^19^ Increasing the number of public datasets and improving data-sharing are crucial to maintaining the field’s momentum in the research of AI.

The priorities for the BSG AI Task Force in the view of participants are identifying research priorities in AI, guidelines for adopting AI devices in clinical practice and supporting the delivery of multicentre trials. Most of the research to date is limited to computer vision, but this is a narrow application of AI. Given the abundance of text used in clinical practice, it may have a far greater reach and potential with other applications such as NLP. The Task Force should identify research priorities to help guide research to where it can best improve patient care and maximise the potential of AI. Developing guidelines for adopting AI in clinical practice was identified as the main barrier to its clinical adoption and a high priority for the Task Force, emphasising its paramount importance. Concerns relating to accountability for the wrong diagnoses and bias of algorithms should be addressed in these guidelines. While gastroenterology is at the forefront of clinical studies of AI, there are still only a limited number of clinical studies with the majority of these single centres.^2 20^ Support from the BSG AI Task Force to deliver multicentre trials would allow a more robust evaluation of the generalisability of AI models.

There are several limitations to this study. As with any voluntary survey, participants who choose to engage with the survey can bias the results. Participants from London made up more than half of the cohort. This is potentially due to AI research mainly being carried out in teaching centres, which is most densely populated in London. The selection of responses in the survey was also limited to those questions decided by the survey developers. The Likert scale also limits participants to categorical responses, which means that we could not measure the true attitude of participants. Furthermore, our cohort represents a small proportion of the UK gastroenterology and endoscopy community, which may not represent the community as a whole.

## Conclusion

This survey of UK gastroenterologists and endoscopists identified some of the perceived benefits, challenges, and barriers to applying AI in clinical practice and AI research. The BSG AI Task Force should consider identifying research priorities, guidelines for adopting AI devices in clinical practice and supporting the delivery of multicentre clinical trials.

10.1136/flgastro-2021-101994.supp1 Supplementary data

## Data Availability

All data relevant to the study are included in the article or uploaded as supplemental information.
