# Supplementary figures and images for: Survey on the perceptions of UK gastroenterologists and endoscopists to artificial intelligence
Source: Frontline Gastroenterol. 2022 Jan 17;13(5):423–9. doi: 10.1136/flgastro-2021-101994 (PMC9380773; doi:10.1136/flgastro-2021-101994)

Which region of the UK do you work in?

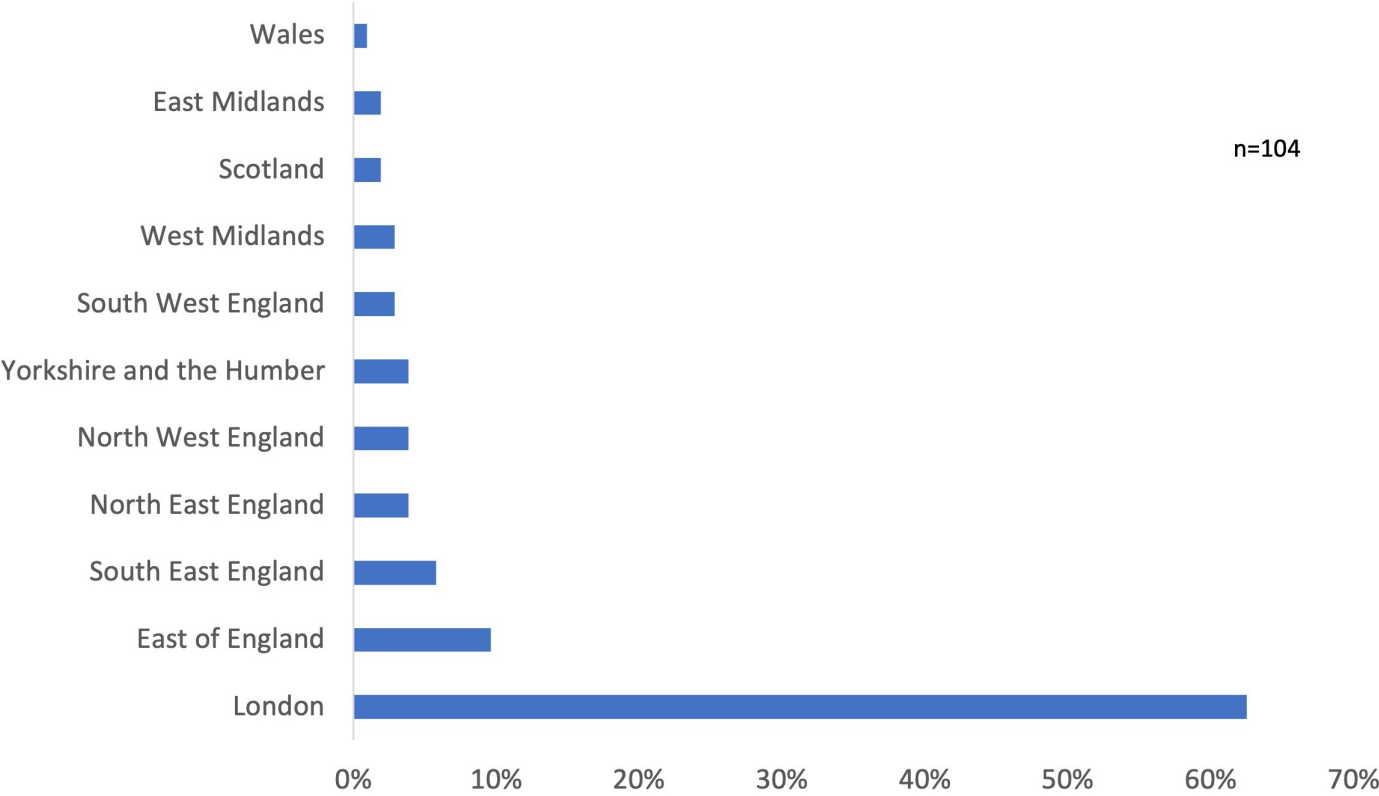

Supplement: Supplementary data [file flgastro-2021-101994supp002.pdf]

### How much speciality experience do you have?

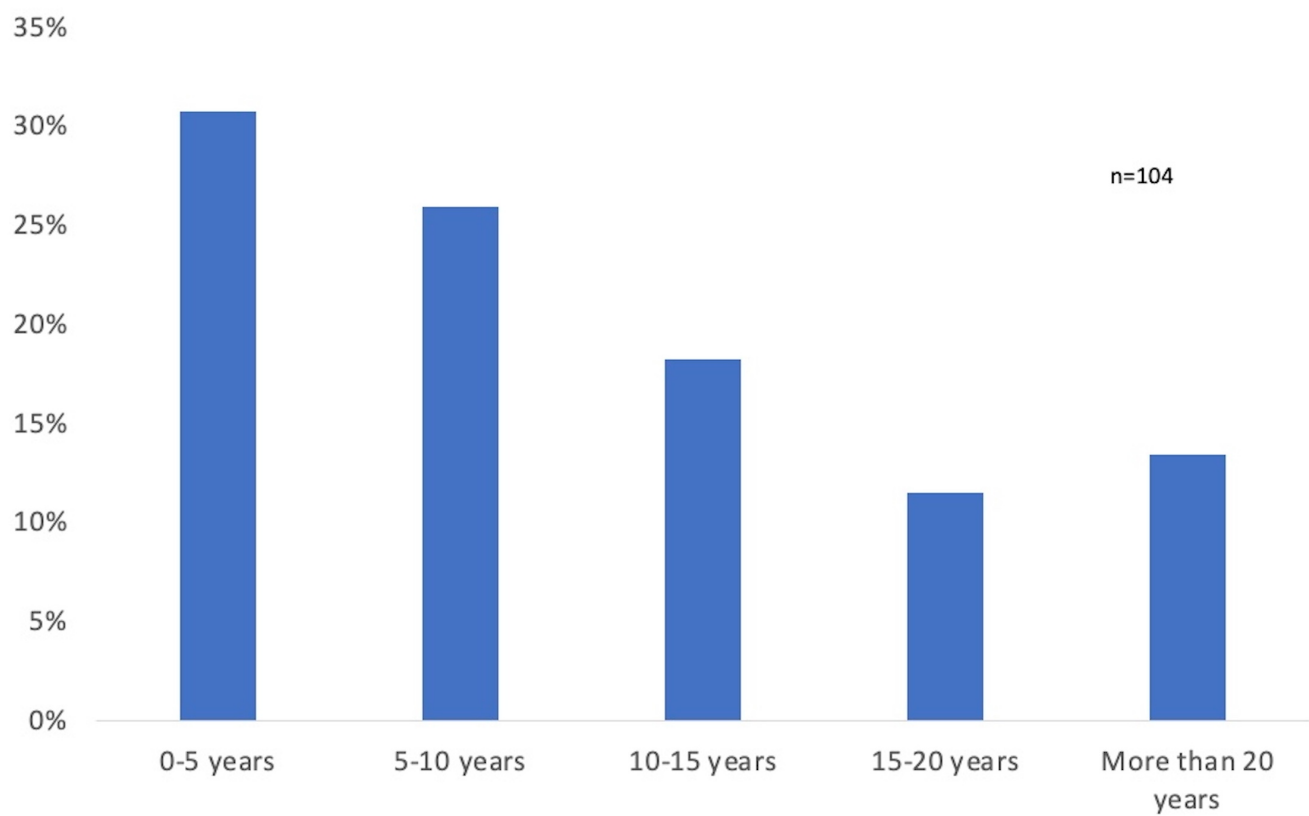

Supplement: Supplementary data [file flgastro-2021-101994supp003.pdf]

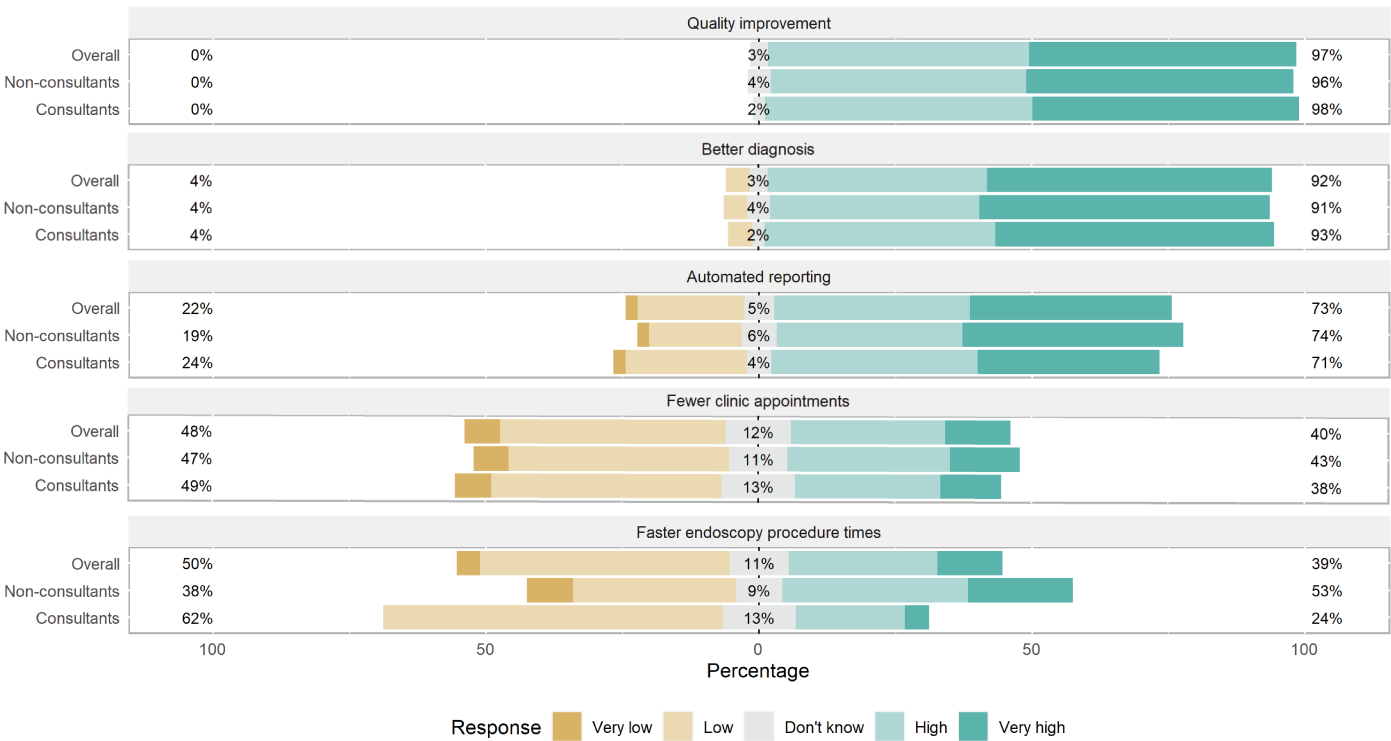

Supplement: Supplementary data [file flgastro-2021-101994supp004.pdf]

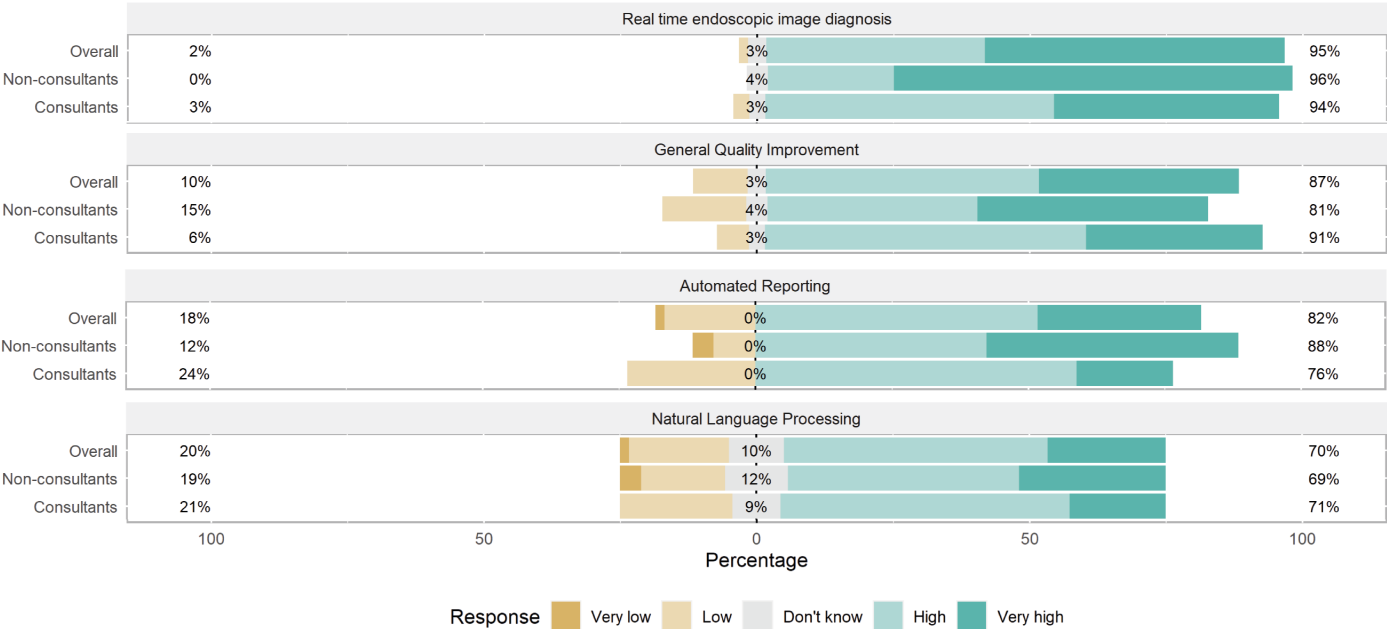

Supplement: Supplementary data [file flgastro-2021-101994supp005.pdf]
