# Supplementary material for: Survey on the perceptions of UK gastroenterologists and endoscopists to artificial intelligence
Source: Frontline Gastroenterol. 2022 Jan 17;13(5):423–9. doi: 10.1136/flgastro-2021-101994 (PMC9380773; doi:10.1136/flgastro-2021-101994)
Supplement: Supplementary data [file flgastro-2021-101994supp001.pdf]

SUPPLEMENTARY SECTION

Supplementary Figure 1: Horizontal bar-chart of participants region of work

Supplementary Figure 2: Vertical bar-chart of participants experience in their speciality

Supplementary Figure 3: Diverging stacked bar chart of the most significant benefits that AI might eventually bring to gastroenterology

Supplementary Figure 4: Participants response to the priorities of AI research
